# Psychometric Properties of an Instrument to Measure Facilitators and Barriers to Nurses’ Participation in Continuing Education Programs

**DOI:** 10.5539/gjhs.v6n5p219

**Published:** 2014-06-11

**Authors:** Zeinab Hamzehgardeshi, Zohreh Shahhosseini

**Affiliations:** 1 Department of Reproductive Health, Nasibeh Nursing and Midwifery Faculty, Mazandaran University of Medical Sciences, Sari, Iran

**Keywords:** continuing education, instrument development, nursing, psychometry

## Abstract

**Background:**

Continuing education programs are one of the professional principles in health-related disciplines, including nursing. The aim of this study was to develop an instrument measuring facilitators and barriers to nurses’ participation in continuing education programs.

**Methods:**

In the first phase, the items generated for the instrument were drawn from a comprehensive literature review along with a polling of experts. Then the psychometric properties were measured.

**Results:**

A Scale-Level Content Validity Index of 0.90 for the primary instrument with 36 items was obtained. The factor structure of inventory was identified by undertaking a Principal Component Analysis in a sample of 361 nurses. Three factors were extracted with a total variance account of 62.67%. Reliability was demonstrated with Cronbach’s alpha coefficient = 0.92. Consistency of instrument was established with test-retest reliability (Intra Cluster Correlation = 0.93, P<0.001).

**Conclusion:**

The major focus of this study was to develop a locally sensitive instrument to assess the facilitators and barriers to Iranian nurses’ participation in continuing education programs.

## 1. Introduction

Continuing Education (CE) is a process that prepares the staff members for improvement and better efficacy in current or future positions, modifies their thinking and action, and furnishes them with professional information they need to achieve organizational goals (Anwar & Batty, 2007; Shojania et al., 2012; Fairchild et al., 2013; Ni et al., 2013). It’s one of the modern strategies to maintain and elevate knowledge in medical community, which in turn elevates the health status of the society. Studies show that knowledge gained through basic professional education has a half-life of 2.5 years, and needs to be updated at the end of this period (Happell, 2004; Chong et al., 2011). Moreover, such training will be expired 5 years after graduation, so lack of CE can lead to poor services to patients (Chong et al., 2011).

CE is one of the principles in medical sciences including nursing. Previous studies have shown nurses attend CE for several personal, professional and organizational reasons included: enhancing professional knowledge, change of routines, improvement in professional success as well as critical thinking, decision making and gaining professional credit (Kristjanson & Scanlan, 1989; Waddell, 1993; Chong et al., 2011). Other reasons included: improvement in professional skills and personal abilities for serving people, personal interests, job security, professional commitment and need to update information (DeSilets 1994; Ebrahimi et al., 2012). Also, supervisor’s support, availability of CE programs and peer encouragement are other effective factors for attending CE from point of views of these groups (Glass Jr & Todd-Atkinson, 1999; Nsemo, John et al., 2013). On the other hand, the literature review have shown huge expenses, time consumption, unawareness of provided CE programs, lack of managers’ support, numerous assigned duties, shortage of nursing staff and poor evaluation system for nurses responsibilities are among the reasons why they allocate little time to CE (Penz et al., 2007; Schweitzer & Krassa, 2010; Chong et al., 2011).

In Iran, nurses ‘participation is mandate in CE and some studies conducted in this country to assess different aspects of CE programs. For example the majority of nurses found the information has been taught in CE courses irrelevant to the wards they worked in, and 60% of them were against this type of education (Ebadi et al., 2007; Ebrahimi et al., 2012). Since several factors affect nurses’ participation in CE, and that their participation in CE affects patients and community health status, it is essential to know facilitators and barriers of nurses’ participation in CE programs and plan accordingly.

In order to carry out research on the above aspects, valid and reliable instruments for measuring facilitators and barriers to nurses’ participation in CE are needed. Now it is accepted that instruments should be culturally sensitive and in accordance with each country’s context (Hilton & Skrutkowski 2002; Maneesriwongul & Dixon, 2004). So, an instrument, namely Iranian Nurses’ Motivation for Continuing Education Inventory (INMCEI) was developed for the purpose of this study.

## 2. Methods

### 2.1 Setting and Data Collection

This was a mixed method study using both qualitative and quantitative approaches. It was performed in two stages during October 2012 to April 2013 in Sari city, North of Iran. At first, a comprehensive literature review on the available surveys measuring facilitators and barriers to nurses ‘participation in CE and opinions of experts was conducted to develop a pool of items for the instrument. In this step, opinions of 10 faculty members of a medical sciences university in Sari, in the area of nursing, continuous education, and instrument development through face-to-face semi-structured interviews were adopted. This allowed the researcher to explain clearly the aims of the study and their expectations of the experts. In this regard, experts were encouraged to provide comments and recommendations concerning any aspect of the INMCEI.

Once the design of the items was completed, the instrument was assessed in terms of face validity. To assess face validity, other 10 experts in the areas mentioned above and 20 nurses from health care centers and educational hospitals in Sari city assessed the significance of the statements in INMCEI. The assessment was based on the item impact method. This method selects items that are most frequently perceived as important by participants on a 5-point scale. The item impact was determined from the proportion of participants who identified it as important and the mean importance score attributed to this item (impact score = frequency × importance). Each statement was accepted if the impact score was ≥ 1.5 (Lacasse et al., 2002). Also to assess face validity, the experts were asked to assess the statements in INMCEI for clarity and fluency and to evaluate item wording, response format, and instrument length.

In next step, a content validity rating form was prepared and given to 12 faculty members of a medical sciences university in Iran to evaluate the instrument. The assessment was based on Content Validity Ratio (CVR) and Content Validity Index (CVI). One widely used method of measuring CVR was developed by Lawshe. It is essentially a method for gauging agreement among raters regarding how essential a particular item is. The experts scored the essentiality of each statement based on “essential”, “useful, but not essential” or “not necessary”. The score for CVR for each statement was calculated by dividing the number of experts who scored each item essential minus total number of experts by the half of total number of experts. The statement was accepted if the CVR was ≥0.56 (Lawshe 1975). For measuring CVI, the experts scored the relevancy, clarity, and simplicity of each statement in INMCEI using a Likert-type scale ranging from 1 to 4 according to Waltz and Bausell index^.^ (Waltz & Bausell, 1981). In this regard, the score for CVI for each statement was calculated by dividing the number of agreed experts (who scored 3 and 4 in the Likert scale) by the total number of experts. The statement was accepted if the CVI was≥0.79 (Hyrkäs et al., 2003).

Construct validity of INMCEI was assessed using exploratory factor analysis method to explore the categories of statements (as the variables) with the highest relativity. The required number of respondents for factor analysis was 3–10 for each statement (Munro, 2005). In this step, 361 nurses were recruited by simple random sampling by assistance of random number table from those who were attended in CE programs which were provided as a routine scheduled project. The Kaiser–Meyer–Olkin test was used to assess the sampling adequacy.

Internal consistency and test–retest reliability methods were used to assess the reliability of INMCEI. Internal consistency reflecting the extent to which each item measures the same construct by creating an average of all possible inter-item correlations by using Cronbach’s alpha coefficient (Bland & Altman, 1997; DeVon et al., 2007). It was calculated in a sample of 20 nurses which were selected by convenience sampling method. Also stability of INMCEI was assessed using test–retest reliability measurement. In this step, the same nurses mentioned above completed the INMCEI forms twice with 2-week interval. The intra-cluster correlation index was used to compare scores of test–retest of INMCEI (Burns & Grove, 2010).

### 2.2 Data Analysis

Finally, the collected data were fed into the Statistical Package for Social Sciences for Windows version 13.0 (SPSS Inc., Chicago, IL, USA) for further analysis.

## 3. Results

Extensive literature reviews on facilitators and barriers to nurses participation in CE and opinions of experts were used to make the preliminary tool with 36 statements (20 statements in area of facilitators and 16 statements in area of barriers) in a 5 level Likert scale (Very important, Moderately important, Somewhat important, Slightly important and Not at all important).

In assessment of face validity based on impact score, as all of the statements gained at least 1.5 score or more, none of them were not omitted. But in assessment of CVR of the instrument, two statements (one in area of facilitators and another one in area of barriers) with score less than 0.56 were omitted. In addition, in assessment of CVI, other two statements (in area of barriers) with score less than 0.79 were omitted too and the statements of the questionnaire reduced to 32. In this way, a Scale-Level Content Validity Index of 0.90 for the instrument was obtained. Besides, researchers attempted to make a correct, reasonable and clear writing of statements based on experts’ opinions to make face validity for the instrument.

In construct validity of INMCEI, after applying factor extraction, a Kaiser-Meyer-Olkin Measure of Sampling Adequacy = 0.93 was obtained which is an acceptable criterion (Munro, 2005) and meant that factor analysis was an appropriate strategy. Bartlett’s Test of Sphericity was also shown to be statistically significant, given Chi Square = 954.71, (*P*<0.001). This indicated that the correlation matrix was not an identity matrix. With principal component analysis, five factors with an eigenvalue of greater than 1 were determined. Because of the low explain ability of the final created factors, and in order to make the instrument simpler and more explainable, exploratory factor analysis was performed again with a limitation in factors’ extraction to three factors, and varimax rotation method was run. Varimax rotation is an orthogonal rotation method usually used for making the construct factors simple and explainable. At this stage, a minimum point of 0.35 was considered as the acceptable minimum factor loading for keeping a statement in an extracted factor following factor analysis. These three factors could explain 62.67% of the variance (Table 1).

**Table 1:** Results of principal components analysis-factors and variance explained Total variance explained

Component	Extraction sums of squared loadings					Rotation sums of squared loadings		
	Total	% of variance		Cumulative %		Total	% of variance	Cumulative %
1	10.03	31.37		31.37		9.72	30.40	30.40
2	8.44	26.39		57.76		6.29	19.67	50.07
3	1.57	4.90		62.67		4.31	12.59	62.67

Extraction method: principal component analysis.

Our result showed facilitator factors were loaded under factor one, which was named incentives’ factors (statements 1-15) and barrier factors were loaded under factors two and three, which was named extra-organizational obstacles (statements 16-27 and 30-32) and intra-organizational obstacles (statements 21, 22, 24 and 26-32) respectively (Table 2). As the results demonstrated, there were some statements which could include in more than one construct. These statements were considered for a construct, based on their factor load and meaning. Therefore, statements 21, 22, 24, 26 and 32 were considered for factor two and statements 27, 30 and 31 were considered for factor three. So, finally extra and intra organizational obstacles included 12 and 5 statements respectively.

**Table 2:** Rotated matrix of an instrument to measure facilitators and barriers to nurses’ participation in CE programs based on principal component analysis and varimax rotation

Statements		1	2	3

		incentives’ factors	extra-organizational obstacles	Intra-organizational obstacles
1	Increase my competency	0.88		
2	Give quality care to patients	0.86		
3	Improve my skill in clinical practice	0.85		
4	Update my knowledge	0.85		
5	Improve my teaching skills	0.84		
6	Obtain knowledge to achieve personal status	0.82		
7	Improve my communication skills	0.81		
8	Improve my decision making skills	0.81		
9	Prevent myself from getting bored with routine	0.79		
10	Improve my research skills	0.79		
11	Adhere to hospital policy	0.78		
12	Boost my self-esteem	0.77		
13	Improve my management skills	0.76		
14	Be more critical in providing nursing care	0.72		
15	Gain more paper qualification	0.52		
16	Emotional stress		0.82	
17	Poor interaction of CE programs’ staff		0.80	
18	Negative experiences with previous CE programs		0.78	
19	Poor physical health		0.71	
20	Domestic responsibilities		0.68	
21	Poor scheduling of CE programs		0.66	0.37
22	Lack of relevant CE programs		0.64	0.35
23	Time constraints		0.64	
24	Geographic distance		0.62	0.40
25	Lack of family support		0.59	
26	Lack of information about CE programs		0.56	0.50
27	Needs satisfied by on the job training		0.55	0.53
28	Lack of supervisors’ support			0.86
29	Lack of peers’ support			0.83
30	Lack of organizational support		0.36	0.81
31	Work commitments		0.39	0.56
32	Cost of courses		0.42	0.56

Reliability analysis was performed based on Cronbach’s alpha coefficient; an important and widely used measure for assessing the internal consistency of a set of items (Cortina 1993). Our results showed INMCEI had an acceptable internal consistency (Gliem and Gliem 2003). It was obtained 0.92 for whole of INMCEI and for the subscales of the instrument including: incentives’ factors (0.96), extra-organizational obstacles (0.94) and intra-organizational obstacles (0.93). Intra Cluster Correlation Coefficient = 0.93 was obtained to assess stability of INMCEI following test re-test reliability (*P*<0.001). Besides, Wilcoxon Signed Rank Test demonstrated no significant differences between the scores of test retest of INMCEI (*P*>0.05).

## 4. Discussion

Participation in CE programs among health care staffs importantly determined the quality of health care services. The authors engaged in the process described herein to develop INMCEI, which its psychometric properties were carefully evaluated, as the first local, culturally-sensitive inventory to assess facilitators and barriers to Iranian nurses’ participation in CE programs. It seems that knowing the factors which can influence nurses’ participation in CE programs, as one of the most important health care providers, is increasingly critical to maintain and improve their knowledge and professional skills.

Content validity of INMCEI was established by assistance of academic members’ expert for evaluating the instrument and in order to obtain the most appropriate item content. This method is one of the best ways to develop an evidence based questionnaire with appropriate content (Rubio et al., 2003; Makhoul et al., 2011).

Construct validity of the INMCEI using factor analysis showed facilitator factors in point of view of Iranian nurses, accounted for near half of the total variance of developed instrument. This issue could be an important issue for managers and policy makers in field of CE programs that promoting facilitators should be considered as important as reducing barriers. Our result in line of other studies showed increasing professional skills and competency in order to provide better health care services for people are from most important incentives for nurses’ participation in CE programs. This issue may root in humanitarian nature of nursing as stated that desire to help and care for others are the reasons for choosing nursing as a major (Ditommaso, 2003; Cho et al., 2010).

Nurses in this study agree that extra- organizational barriers were more obstacles to their participation in CE programs compared to intra-organizational ones. Also some of these barriers have reported in similar studies, but some of them were less published before. In this way, our participants stated that psycho-emotional stress is one of the important reasons why they allocate little time to CE programs. Some studies have shown job dissatisfaction; staff and health care team’s negative attitudes towards nurses and poor social position of nurses are from the major challenges in Iranian nursing profession (Farsi et al., 2010; Zarea et al., 2012). This factor alongside by domestic responsibilities and lack of family support as well as poor management of provided CE programs shape the most important barriers to nurses participation in CE as extra- organizational barriers explained 19.67% of 30.37% of total variances related to barrier factors. Although it’s noticeable that reducing intra- organizational obstacles like: lack of peers and managers’ support as well as shortage of nurses staff should be considered to improve CE programs efficacy (Penz et al., 2007; Chong et al., 2011)^.^

In conclusion this study used both quantitative and qualitative methods to develop an instrument in field of CE. The content validity, construct validity, and reliability of the 32-item inventory were ensured by expert review, factor analysis and internal consistency, respectively. The INMCEI was validated in a Persian language version and a translated version in any other language would also require appropriate validation. The results of this study may provide insightful information to CE managers in field of nursing. It seems development a valid and reliable instrument like INMCEI could be considered as a pilot project to identifying major factors to develop more effective CE programs.

From limitation of our study was that the items generated in this instrument based on the literature review and experts’ opinions. If some interviews with nurses had been done, it might be possible other items had been emerged.
